# AKIN10 delays flowering by inactivating IDD8 transcription factor through protein phosphorylation in *Arabidopsis*

**DOI:** 10.1186/s12870-015-0503-8

**Published:** 2015-05-01

**Authors:** Eun-Young Jeong, Pil Joon Seo, Je Chang Woo, Chung-Mo Park

**Affiliations:** Department of Chemistry, Seoul National University, Seoul, 151-742 South Korea; Department of Biological Science, Mokpo National University, Jeonnam, 534-729 South Korea; Plant Genomics and Breeding Institute, Seoul National University, Seoul, 151-742 South Korea

**Keywords:** *Arabidopsis thaliana*, Flowering time, Sugar metabolism, IDD8, SnRK1, AKIN10, Protein phosphorylation

## Abstract

**Background:**

Sugar plays a central role as a source of carbon metabolism and energy production and a signaling molecule in diverse growth and developmental processes and environmental adaptation in plants. It is known that sugar metabolism and allocation between different physiological functions is intimately associated with flowering transition in many plant species. The INDETERMINATE DOMAIN (IDD)-containing transcription factor IDD8 regulates flowering time by modulating sugar metabolism and transport under sugar-limiting conditions in *Arabidopsis*. Meanwhile, it has been reported that SUCROSE NONFERMENTING-1-RELATED PROTEIN KINASE 1 (SnRK1), which acts as a sensor of cellular energy metabolism, is activated by sugar deprivation. Notably, *SnRK1*-overexpressing plants and *IDD8*-deficient mutants exhibit similar phenotypes, including delayed flowering, suggesting that SnRK1 is involved in the IDD8-mediated metabolic control of flowering.

**Results:**

We examined whether the sugar deprivation-sensing SnRK1 is functionally associated with IDD8 in flowering time control through biochemical and molecular genetic approaches. Overproduction of AKIN10, the catalytic subunit of SnRK1, delayed flowering in *Arabidopsis*, as was observed in *IDD8*-deficient *idd8-3* mutant. We found that AKIN10 interacts with IDD8 in the nucleus. Consequently, AKIN10 phosphorylates IDD8 primarily at two serine (Ser) residues, Ser-178 and Ser-182, which reside in the fourth zinc finger (ZF) domain that mediates DNA binding and protein-protein interactions. AKIN10-mediated phosphorylation did not affect the subcellular localization and DNA-binding property of IDD8. Instead, the transcriptional activation activity of the phosphorylated IDD8 was significantly reduced. Together, these observations indicate that AKIN10 antagonizes the IDD8 function in flowering time control, a notion that is consistent with the delayed flowering phenotypes of *AKIN10*-overexpressing plants and *idd8-3* mutant.

**Conclusion:**

Our data show that SnRK1 and its substrate IDD8 constitute a sugar metabolic pathway that mediates the timing of flowering under sugar deprivation conditions. In this signaling scheme, the SnRK1 signals are directly integrated into the IDD8-mediated gene regulatory network that governs flowering transition in response to fluctuations in sugar metabolism, further supporting the metabolic control of flowering.

**Electronic supplementary material:**

The online version of this article (doi:10.1186/s12870-015-0503-8) contains supplementary material, which is available to authorized users.

## Background

Appropriate timing of flowering is important for propagation and reproductive success in plants. Therefore, flowering time is precisely regulated through the coordinated actions of endogenous developmental cues, such as plant aging and gibberellic acid (GA), and environmental signals, including changes in the length of day or photoperiod and temperature [1-3]. The floral inductive and repressible signals are transduced through well-established flowering genetic pathways, such as photoperiod, vernalization, GA, autonomous, and thermosensory pathways [1,4], and converge at the floral promoters *FLOWERING LOCUS T* (*FT*) and *SUPPRESSOR OF CONSTANS OVEREXPRESSION 1* (*SOC1*) and the floral repressor *FLOWERING LOCUS C* (*FLC*) [4,5].

Accumulating evidence support that sugar metabolism and distribution is intimately associated with flowering time control in many plant species [1,6]. Plants that are defective in sugar biosynthesis and metabolism exhibit alterations in developmental traits and flowering time [6,7]. It is widely perceived that plants do not flower even under photo-inductive conditions until they accumulate enough sugar reserves for the induction of flowering [6-8], which is consistent with the observations that low-starch-containing mutants, such as *pgm1* and *pgi*, exhibit retarded growth and delayed flowering [9,10]. Endogenous sugar levels are directly linked with photosynthetic carbon assimilation [6], indicating that photosynthetic activity also influences flowering transition [11].

While the effects of sugar on flowering time have been widely documented in many plant species, it is still unclear how sugar regulates the timing of flowering. In some cases, sugar promotes flowering, whereas flowering is inhibited in other cases, depending on different plant genotypes and growth conditions [8,12]. The functional ambiguity of sugar in flowering time control reflects the complexity of sugar homeostasis, which is attributed to the combined regulation of biosynthesis, degradation, and distribution in different plant tissues [6,8,12]. Sugar transport also plays a role in flowering time control. *Arabidopsis* mutants that have mutations in *SUCROSE TRANSPORTER9* (*AtSUC9*) gene exhibits early flowering under short days [13]. It has been suggested that AtSUC9 mediates the directional transport of sugar from the phloem to the sink organs and thus reduces sugar transport to the shoot apical meristem. It is also known that down-regulation of *StSUT4* gene in potato promotes flowering [14], supporting the linkage between sugar transport and flowering induction.

Roles of sucrose-regulated protein kinases and trehalose-6-phosphate (T6P) have been studied in linking sugar metabolism with flowering transition [15-17]. The T6P pathway has been shown to function upstream of the floral integrator FT in the leaves and regulates a flowering pathway that involves microRNA156 and SQUAMOSA PROMOTER-BINDING PROTEIN-LIKE (SPL) proteins in the shoot apical meristem [17], supporting a linkage between sugar and a distinct flowering pathway. In addition, it has been shown that photoperiodic control of sugar metabolism is associated with flowering induction in *Arabidopsis* and soybean [18]. Notably, CONSTANS (CO), which is a central regulator of photoperiodic flowering in *Arabidopsis* [4], plays a key role in the signaling pathway by regulating the expression of genes that are involved in sugar metabolism [19], providing a direct evidence that sugar metabolism is linked with photoperiod flowering.

The INDETERMINATE DOMAIN (IDD)-containing transcription factor IDD8 has been shown to regulate photoperiodic flowering under sugar deprivation conditions [20]. Whereas *IDD8*-defective *idd8* mutants exhibit late flowering, *IDD8*-overexpressing plants exhibit early flowering. The expression of *SUC* and sucrose synthase (*SUS*) genes is altered in the transgenic plants and *idd8* mutants. It has been reported that IDD8 regulates the *SUS* genes by directly binding to the gene promoters [20]. Moreover, the *SUS* genes are regulated by photoperiods, indicating that IDD8 regulation of sucrose metabolism and transport is associated with photoperiodic flowering. However, it is not known how sugar deprivation signals regulate IDD8 activity at the molecular level.

It is notable that T6P inhibits the activity of the Sucrose-non-fermenting1 (Snf1)-related kinase 1 (SnRK1) in sugar metabolic control of flowering [21]. SnRK1 is a serine/threonine protein kinase that is homologous to yeast Snf1 and animal AMP-dependent protein kinase 1 (AMPK1) kinases [22,23]. SnRK1/Snf1/AMPK acts as a metabolic sensor in eukaryotes and is activated under energy deprivation conditions [24,25]. In particular, *snrk1* knockdown plants exhibit early flowering, whereas *SnRK1* overexpression delays flowering [24,26]. In addition, SnRK1 is activated, but IDD8 is inactivated under sugar-limiting conditions, suggesting that SnRK1 and IDD8 are functionally interrelated in the sugar metabolic control of flowering.

In this work, we found that AKIN10, the catalytic α-subunit of SnRK1 kinases [27], phosphorylates IDD8 in the nucleus. While AKIN10-mediated phosphorylation did not affect the nuclear location and DNA-binding property of IDD8, it significantly reduced the transcriptional activation activity of IDD8. These results demonstrate that low-sugar levels trigger the SnRK1-mediated inactivation of IDD8 through protein phosphorylation, leading to delay of flowering. The SnRK1-IDD8 module would also be involved in the timing of flowering under abiotic stress conditions, which limit photosynthetic activity and disturb sugar metabolism in plants [28,29].

## Results

### *idd8-3* and *AKIN10-*overexpresser exhibit delayed flowering under long days

As an initial step to investigate the functional relationship between AKIN10 and IDD8 in flowering time control, we compared the flowering phenotypes of *Arabidopsis* plants that have altered expression of *IDD8* and *AKIN10* genes. T-DNA insertional mutants of *AKIN10* and *AKIN11* genes (*akin10-1* and *akin11-1*, respectively) were obtained from the *Arabidopsis* Biological Resource Center (ABRC, Ohio state university, OH). Gene expression analysis revealed that they are loss-of-function mutants (Additional file 1). We also produced transgenic plants overexpressing either *AKIN10* or *AKIN11* gene under the control of the cauliflower mosaic virus (CaMV) 35S promoter, resulting in *10*-ox or *11*-ox, respectively (Additional file 2). We similarly produced transgenic plants overexpressing *IDD8*, resulting in *8*-ox.

**Figure 1:**
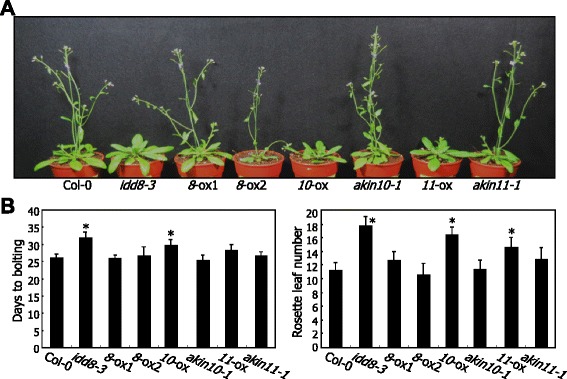
*AKIN10* overexpression delays flowering. Plants were grown in soil under LDs for 6 weeks before taking photographs **(A)**. Flowering times were measured by counting the days to bolting and rosette leaf numbers at bolting (**B**, left and right panels, respectively). Transgenic plants overexpressing *IDD8* (*8*-ox1 and *8*-ox2), *AKIN10* (*10*-ox), and *AKIN11* (*11*-ox) and their gene knockout mutants were analyzed. The countings of approximately 20 plants were averaged and statistically analyzed using Student *t*-test (*P < 0.01, difference from col-0). Bars indicate standard error of the mean.

We examined the flowering phenotypes of the plants grown under long days (LDs, 16-h light and 8-h dark) by counting the numbers of rosette leaves at bolting and the days to bolting. The *8*-ox plants and the *akin10-1* and *akin11-1* mutants did not exhibit any discernible flowering phenotypes under our assay conditions (Figures 1A and 1B). In contrast, the *10*-ox and *11*-ox plants exhibited delayed flowering, as observed in *idd8-3* mutant. The delay of flowering time was more prominent in *10-ox* than in *11-ox* (Figure 1B). The similar flowering phenotypes raised a possibility that loss of IDD8 function is related with overproduction of AKIN10 and AKIN11 in regulating flowering time. In support of this hypothesis, the expression of *SUS4* and *SUC* genes was suppressed in the *10*-ox plants but up-regulated in the *akin10-1* mutant (Additional file 3), as observed in the *idd8-3* mutant and the *8*-ox plants, respectively [20].

### IDD8 interacts with AKIN10 in the nucleus

On the basis of the similar flowering phenotypes of *idd8-3* mutant and AKIN-overexpressing plants and the biochemical nature of IDD8 transcription factor and SnRK1 kinases, we hypothesized that IDD8 interacts with the SnRK1 kinases.

**Figure 2:**
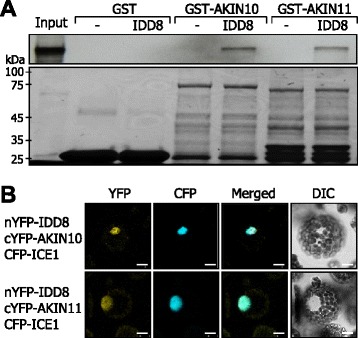
IDD8 interacts with AKIN proteins in the nucleus. **A** *in vitro* pull-down assay. Recombinant GST-AKIN10 and GST-AKIN11 fusion proteins produced in *E. coli* cells and *in vitro* translated, radio-labelled IDD8 polypeptides were used (upper panel). Recombinant GST was used as negative control. The ‘Input’ represents 20% of the labeling reaction. Part of Coomassie Blue-stained gel was displayed as a loading control (lower panel). kDa, kilodalton. **B** BiFC assay. nYFP-IDD8 and cYFP-AKIN fusions and cyan fluorescent protein (CFP)-ICE1 fusion, which was used as a nuclear marker, were coexpressed transiently in *Arabidopsis* protoplasts. IDD8-AKIN interactions were visualized by differential interference contrast (DIC) and fluorescence microscopy. Scale bars, 10 μm.

Yeast two-hybrid assays did not show any positive interactions between IDD8 and AKIN10 (data not shown). We therefore employed *in vitro* pull-down assays using recombinant glutathione S-transferase-AKIN10 (GST-AKIN10) and GST-AKIN11 fusion proteins, which were produced in *E.coli* cells, and ^35^S-labelled IDD8 polypeptides produced by *in vitro* translation. While IDD8 did not interact with GST alone, it strongly interacted with GST fusions of AKIN10 and AKIN11 (Figure 2A). The lack of IDD8-AKIN interactions in yeast cells might be due to an intrinsic property of AKIN proteins, as has been observed previously [27,30].

We also performed bimolecular fluorescence complementation (BiFC) assays to examine whether the IDD8-AKIN interactions occur in plant cells. Coexpression of the N-terminal half of yellow fluorescent protein (YFP) fused to IDD8 (nYFP-IDD8) and the C-terminal half of YFP fused to AKIN10 (cYFP-AKIN10) or AKIN11 (cYFP-AKIN11) in *Arabidopsis* protoplasts revealed that the IDD8-AKIN interactions occur in the nucleus (Figure 2B, Additional file 4), indicating that IDD8 interacts with AKIN proteins *in planta*.

### AKIN10 phosphorylates IDD8

AKIN10 and AKIN11 are the catalytic subunits of SnRK1 kinases [24,27]. Protein phosphorylation is one of the primary biochemical mechanisms that modulate the activities of transcription factors in plants [26,31,32]. We therefore examined whether AKIN proteins phosphorylate IDD8.

**Figure 3:**
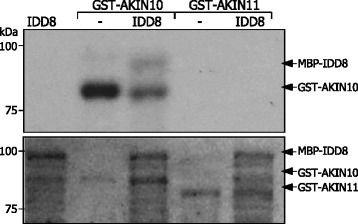
Phosphorylation of IDD8 by AKIN10. The *in vitro* phosphorylation assays were conducted using recombinant GST-AKIN10 and GST-AKIN11 fusion proteins and MBP-IDD8 fusion protein prepared in *E. coli* cells (upper panel). Part of Coomassie Blue-stained gel was displayed as a loading control (lower panel). kDa, kilodalton.

We produced recombinant maltose-binding protein-IDD8 (MBP-IDD8) and GST-AKIN fusion proteins in *E.coli* cells, which were purified by affinity chromatography and immunologically quantified (Additional file 5A). The *in vitro* kinase assays showed that AKIN10 possesses an autophosphorylation activity, while AKIN11 does not (Figure 3). It was also evident that AKIN10, but not AKIN11, phosphorylates IDD8. Although both *10*-ox and *11*-ox plants exhibited delayed flowering (Figure 1) and IDD8 interacts with both AKIN10 and AKIN11, IDD8 may not be directly targeted by AKIN11 at least in controlling flowering time.

**Figure 4:**
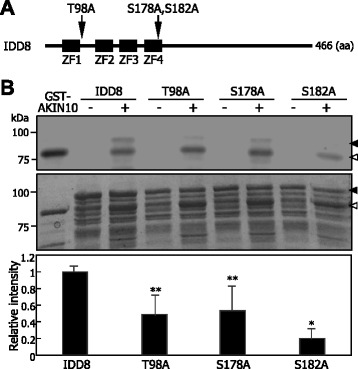
Identification of phosphorylation residues in IDD8. **A** Predicted phosphorylation residues in IDD8. Potential phosphorylation residues were predicted using the NetPhos-based analysis tool (http://www.cbs.dtu.dk/services/NetPhos/). The predicted serine (S) and threonine (T) residues were mutated to alanine (A). ZF, zinc finger. aa, amino acid. **B** *in vitro* phosphorylation assay. The assays were conducted using recombinant GST-AKIN10 and MBP-IDD8 fusion proteins prepared in *E. coli* cells (upper panel). Part of Coomassie Blue-stained gel was displayed as a loading control (middle panel). Black arrowheads indicate IDD8 protein. White arrowheads indicate AKIN10 protein. kDa, kilodalton. The relative intensities of the phosphorylation bands were calculated in comparison to those on Coomassie Blue-stained gel (lower panel). Experimental triplicates were averaged and statistically analyzed using Student *t*-test (*P < 0.01, **P < 0.05, difference from wild-type IDD8). Bars indicate standard error of the mean.

To identify the Ser and Thr residues of IDD8 targeted by AKIN10, we searched for putative target residues using the NetPhos2 algorithm (http://www.cbs.dtu.dk/services/NetPhos/). The computer-assisted analysis identified 18 Ser and 5 Thr residues that were predicted to be phosphorylated by SnRK1. Among the 23 residues, only the sequence contexts around Thr-98, Ser-178, and Ser-182 partially matched to the consensus sequence established for SnRK1 kinases [26] (Additional file 6). The three residues were mutated to alanine, resulting in T98A, S178A, and S182A (Figure 4A), and the mutated IDD8 proteins were prepared as MBP fusions in *E. coli* cells and immunologically quantified (Additional file 5B). The recombinant MBP-IDD8 proteins were then subjected to *in vitro* phosphorylation assays. It was found that the phosphorylation of S182A was significantly reduced by more than 90% compared to that of wild-type IDD8 protein (Figure 4B). In contrast, T98A and S178A were still phosphorylated with a reduction of approximately 50%. Liquid chromatography-tandem mass spectrometry (LC-MS/MS) also supported the notion that S182 is a major site for AKIN10-mediated phosphorylation (Additional file 7).

### AKIN10 does not affect the subcellular localization of IDD8

Protein phosphorylation influences diverse structural and functional aspects of transcription factors, such as protein stability, subcellular localization, and transcriptional activation activity [26,32,33]. It has been reported that AKIN10 regulates the protein stability of the B3-domain-containing transcription factor FUSCA3 (FUS3) during lateral organ development and floral transition [26]. Therefore, a question was how AKIN10-mediated phosphorylation regulates IDD8 function in flowering time control.

We first examined whether protein phosphorylation affects the stability of IDD8 protein using transgenic plants overexpressing *IDD8-MYC* fusion driven by the CaMV 35S promoter in either Col-0 plant or *akin10-1* mutant. The transgenic plants were incubated either in constant light or in complete darkness for 2 days. They were also incubated in the presence of 3-(3,4-dichlorophenyl)-1,1-dimethylurea (DCMU), which is a specific inhibitor of photosynthesis [24], in constant light. IDD8 proteins were then immunologically detected using an anti-MYC antibody. The results showed that in the Col-0 background, the IDD8 levels were reduced in darkness, and the reduction was more prominent in the presence of DCMU (Additional file 8A, upper panel), which is probably due to dark-induced degradation of IDD8 protein. Alternatively, the reduction would be at least in part attributable to the transcriptional suppression of *IDD8* gene by low sugar levels. Notably, the patterns of IDD8 abundance were similarly observed in *akin10-1* background, although the overall levels were lower than those in Col-0 background. Quantitative real-time RT-PCR (qRT-PCR) showed that the levels of *IDD8* transcripts were lower in *akin10-1* background (Additional file 8A, lower left panel). However, the levels of IDD8 protein relative to those of *IDD8* transcripts were similar in Col-0 and *akin10-1* backgrounds (Additional file 8A, lower right panel). Together, these observations indicate that AKIN10 does not affect the stability of IDD8 protein.

We next examined whether AKIN10-mediated phosphorylation influences the subcellular localization of IDD8 by transient expression of a green fluorescent protein (GFP)-IDD8 fusion in *Arabidopsis* protoplasts prepared from Col-0, *akin10-1*, and *10*-ox plants and using transgenic plants overexpressing a *GFP-IDD8* fusion in Col-0 and *10*-ox backgrounds. The roots of the transgenic plants were visualized by fluorescence microscopy. GFP signals were detected predominantly in the nuclei of root cells of both Col-0 and *10*-ox backgrounds (Additional files 8B and C), indicating that the subcellular distribution of IDD8 is not affected by AKIN10-mediated protein phosphorylation.

### AKIN10 inhibits the transcriptional activation activity of IDD8

IDD8 binds directly to *SUS4* gene promoter containing the conserved CTTTTGTCC motif [20]. We therefore asked whether AKIN10 affects the DNA-binding property of IDD8. We performed chromatin immunoprecipitation (ChIP) assays using 35S:*MYC-IDD8* and 35:*MYC-IDD8 akin10-1* plants. IDD8-binding sequence (BS) and non-binding sequence (nBS) within the *SUS4* gene promoter were included in the assays (Additional file 9A). The assays revealed that IDD8 does not bind to nBS sequence (Additional file 9B). In contrast, IDD8 efficiently bound to BS sequence. Notably, IDD8 also bound efficiently to BS sequence in *akin10-1* background, indicating that AKIN10 does not affect the DNA-binding property of IDD8.

**Figure 5:**
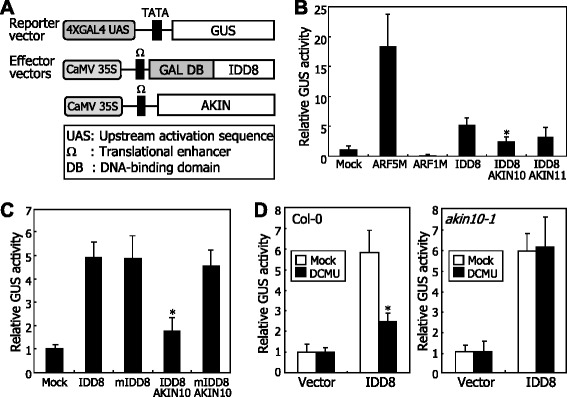
AKIN10 inhibits IDD8 transcription factor activity. **A** Reporter and effector vector constructs. A full-size *IDD8* cDNA was fused in-frame to the 3′ end of GAL4 DNA-binding domain (DB)-coding sequence in the effector vector. **B** SnRK1-mediated inhibition of IDD8 transcriptional activation activity. GAL4 transient expression assays were performed using *Arabidopsis* protoplasts, as described previously [20]. The *Renilla* luciferase gene was used as an internal control to normalize the values in individual assays. ARF5M is a transcriptional activator control. ARF1M is a transcriptional repressor control. Three measurements of GUS activity were averaged and statistically analyzed using Student *t*-test (*P < 0.01, difference from IDD8). Bars indicate standard error of the mean. **C** Transcription factor activity of mutated IDD8. The mutated IDD8 (mIDD8) harbors S178A and S182A substitutions. GUS activity measurements were performed as described in **(B)**. Bars indicate standard error of the mean (*t*-test, *P < 0.01, difference from IDD8). **D** Effects of sugar deprivation on IDD8 transcription factor activity. The GUS reporter and the IDD8 effector vectors were cotransformed into *Arabidopsis* protoplasts that were prepared from either Col-0 plant or *akin10-1* mutant (left and right panels, respectively). The *Arabidopsis* protoplasts were then treated with 20 μM DCMU for 6 h before GUS activity measurements. Three measurements were averaged and statistically analyzed (*t*-test, *P < 0.01, difference from mock). Bars indicate standard error of the mean.

A remaining question was whether AKIN10 affects the transcriptional activation activity of IDD8. To address this question, we performed transient β-galactosidase (GUS) expression assays by coexpressing a series of reporter and effecter vectors in *Arabidopsis* protoplasts (Figure 5A). Notably, AKIN10 reduced the transcriptional activation activity of IDD8 by approximately 65% (Figure 5B). In contrast, AKIN11 reduced the IDD8 activity only slightly, further supporting the notion that AKIN11 is not directly related with IDD8.

The transient GUS expression assays also showed that a mutated IDD8 protein (mIDD8) harboring the S178A and S182A substitutions is transcriptionally active comparable to the wild-type IDD8 protein (Figure 5C). It was notable that whereas AKIN10 reduced the IDD8 activity, it did not affect the mIDD8 activity, indicating that IDD8 phosphorylation by AKIN10 is important for the suppression of the IDD8 activity.

It is known that AKIN10 is activated under low-sugar conditions [25]. We therefore examined the effects of sugar deprivation on IDD8 activity by transient GUS expression assays using *Arabidopsis* protoplasts prepared from Col-0 plants and *akin10-1* mutant. *Arabidopsis* protoplasts were treated with DCMU to mimic sugar deprivation conditions before the assays. It was found that whereas DCMU detectably reduced the IDD8 activity in Col-0 plants, it did not affect the IDD8 activity in *akin10-1* mutant (Figure 5D), demonstrating that AKIN10 suppresses IDD8 activity under sugar deprivation conditions.

### AKIN10-mediated phosphorylation of IDD8 is relevant for flowering time control

**Figure 6:**
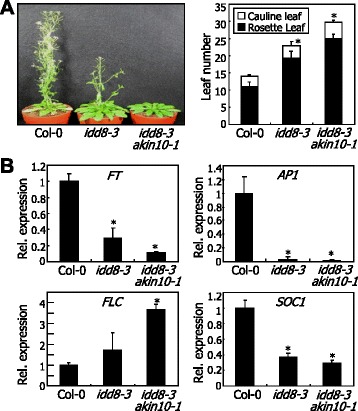
Flowering phenotypes and molecular characterization of *idd8-3 akin10-1* double mutant. The *idd8-3* mutant was crossed with the *akin10-1* mutant, resulting in *idd8-3 akin10-1* double mutant. **A** Flowering phenotypes. Plants were grown in soil under LDs for 6 weeks before taking photographs (left panel). Leaf numbers of 20 plants at bolting were averaged and statistically analyzed using the Student t-test (*P < 0.01, difference from Col-0) (right panel). Bars indicated standard error of the mean. **B** Expression of flowering time genes. Aerial parts of two-week-old plants grown in soil were harvested at zeitgeber time 16 for the extraction of total RNA. Transcript levels were examined by qRT-PCR. Biological triplicates were averaged and statistically analyzed using Student *t*-test (*P < 0.01, difference from Col-0). Bars indicate standard error of the mean.

Our data showed that AKIN10 phosphorylates IDD8 to reduce its transcriptional activation activity in response to sugar deprivation. We next examined whether the phosphorylation of IDD8 by sugar deprivation-activated AKIN10 is functionally relevant for flowering time control. We crossed *idd8-3* with *akin10-1*, resulting in *idd8-3 akin10-1* double mutant (Additional file 10). Flowering time measurements showed that the *idd8-3 akin10-1* double mutant exhibited delayed flowering as observed in the *idd8-3* mutant (Figure 6A). What was unexpected was that the delay of flowering was more severe in the double mutant, suggesting that AKIN10 might target additional flowering time modulators other than IDD8 (see below).

qRT-PCR assays on flowering time genes showed that *FT* gene and its downstream targets *SOC1* and *APPETALA 1* (*AP1*) genes were suppressed in the single and double mutants (Figure 6B), consistent with their delay flowering phenotypes. Notably, the floral repressor *FLC* was significantly induced in the *idd8-3 akin10-1* mutants, which might be related with the severity of delayed flowering in the double mutant (Figure 6A).

**Figure 7:**
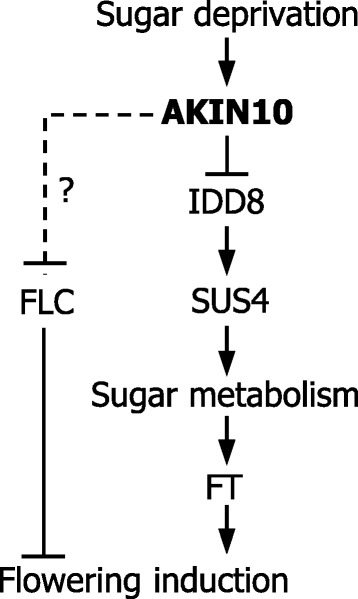
Schematic model of AKIN10 function in flowering time control. Sugar deprivation conditions, which are encountered in early vegetative phase, activate AKIN10 that negatively regulates IDD8 transcription factor. During the reproductive phase transition, increased sugar availability deactivates AKIN10, resulting in flowering transition. It is also likely that AKIN10 negatively regulates FLC function either directly or indirectly via an unidentified regulator of FLC.

Altogether, our data demonstrate that SnRK1 inhibits the transcriptional activation activity of IDD8 transcription factor through protein phosphorylation to delay flowering under low-sugar conditions (Figure 7). This working scenario explains the suppression of IDD8 function under sugar deprivation conditions [20]. We propose that the SnRK1-IDD8 signaling module provides a molecular clue for the long-lasting interest in the metabolic control of flowering in plants.

## Discussion

In this work, we demonstrated that the serine/threonine-specific kinase SnRK1 and its target IDD8 transcription factor constitute a sugar metabolism-mediated flowering pathway. On the basis of molecular characterization of *idd8-3* and *akin10-1* mutants and transgenic plants overexpressing *IDD8* or *AKIN10* genes and biochemical examination of AKIN10-mediated phosphorylation of IDD8, we suggest that the SnRK1 pathway senses fluctuations in sugar metabolism and integrates the metabolic signals into the IDD8-mediated gene regulatory network that regulates flowering time.

There has been a controversy on the molecular nature of *akin10-1* mutant. It has been reported that the *akin10-1* mutant is a null mutant through *AKIN10* gene expression study and immunological detection of AKIN10 proteins using two-dimensional SDS-PAGE [34]. Meanwhile, is has been shown that *AKIN10* gene sequence was amplified and AKIN10 protein was detected in the *akin10-1* mutant [26]. We verified that the *AKIN10* gene is disrupted by the insertion of T-DNA element and it is not expressed in the mutant by PCR-based genotyping and qRT-PCR using different sets of primers. We also found that *SUS4* gene expression is altered in the *akin10-1* mutant that exhibits differential response to DCMU. We believe that *akin10-1* is a loss-of-function mutant. The amplification of *AKIN10* sequence and detection of AKIN10 protein in the previous report would be due to a high sequence similarity among *AKIN* gene members and similar sizes of AKIN proteins in *Arabidopsis*.

### SnRK1-IDD8 module in sugar metabolic control of flowering

Floral transition is one of the most energy-consuming developmental processes in plants. Therefore, it is not surprising that the timing of flowering is closely associated with sugar homeostasis. In view of metabolic control of flowering, it is notable that SnRK1 plays a fundamental role in the developmental process in response to carbon availability [35]. SnRK1 members coordinate diverse transcriptional regulatory networks that stimulate catabolism but suppress anabolism to sustain cellular energy homeostasis under stressful conditions [24,35,36]. While the roles of SnRK1 members have been reported in various cellular responses, only a few substrates have been identified so far.

One of the best characterized targets is the FUS3 transcription factor, which regulates seed maturation in *Arabidopsis* [37]. It has been shown that AKIN10-mediated phosphorylation enhances the FUS3 activity by improving its protein stability [26]. Accordingly, FUS3 is involved in the SnRK1-mediated control of developmental phase transitions. Molecular genetic assays have shown that the *fus3-3* mutation partially rescued the delayed flowering of *AKIN10*-overexpressing plants [26]. However, the *FUS3* gene is not detectably induced during the vegetative-to-reproductive phase transition, and the flowering phenotype of the *fus3-3* mutant is similar to that of control plants [26,37]. Together with the partial recovery of the flowering phenotype of *AKIN10*-overexpressing plants by the *fus3-3* mutation, it has been suggested that the SnRK1-mediated metabolic signals are not solely mediated by FUS3 in regulating flowering time control [26].

In this study, we demonstrated that AKIN10, which is the catalytic subunit of SnRK1 kinases [27], negatively regulates the transcriptional activation activity of IDD8 transcription factor through protein phosphorylation. While our data strongly support that IDD8 is phosphorylated by AKIN10, it is still possible that other kinases would also phosphorylate IDD8, assuming the roles of IDD8 in sugar homeostasis and flowering time control [20, see below]. IDD8 induces *SUS4* gene by directly binding to the gene promoter, leading to the promotion of photoperiodic flowering [20]. The *IDD8* gene is suppressed by sugar deprivation [27]. Together with the previous observations, our data show that SnRK1 mediates the inactivation of IDD8 in flowering time control under low-sugar conditions. It is currently unclear whether IDD8 is functionally connected with FUS3 in the process of sensing sugar metabolic status by SnRK1.

### SnRK1-mediated inactivation of IDD8 activity

Protein phosphorylation influences the activity of transcription factors through diverse mechanisms, such as modulation of their nucleo-cytoplasmic distributions, DNA-binding properties, and protein stabilities and modification of their interactions with other regulatory proteins [33,38,39]. AKIN10 does not affect the nuclear localization and DNA binding of IDD8. The protein stability of IDD8 is also unaffected by protein phosphorylation. Instead, AKIN10-mediated phosphorylation inhibits the transcriptional activation activity of IDD8.

A critical question is how protein phosphorylation affects the IDD8 activity. We found that AKIN10 phosphorylates IDD8 primarily at Ser-182, which resides in the fourth ZF domain. IDD8 has four copies of ZF domains, which are known to mediate DNA binding and protein-protein interactions [40,41]. It has been reported that a central amino acid sequence region of IDD8, which includes residues 171–320 and thus harbors the fourth ZF domain, contains a potential transcriptional activation domain [20]. It has been suggested that the fourth ZF domain mediates the interactions of IDD transcription factors with other interacting partners in regulating the expression of target genes [20]. We suspect that a similar regulatory scheme is applicable to the inhibition of the IDD8 activity by AKIN10: AKIN10 might inhibit the interaction of IDD8 with other regulatory proteins by phosphorylating the critical residues in the fourth ZF domain. In this regard, it will be interesting to investigate whether FUS3 interacts with IDD8 through the fourth ZF domain.

### Additional roles of SnRK1-IDD8 module beyond metabolic control of flowering?

Plant adaptation responses to stressful conditions, such as drought, high salinity, and extreme temperatures, frequently accompany alterations in sugar metabolism and transport [42-44]. It has been known that SnRK1 kinases are associated with plant responses to environmental stress conditions by linking cellular energy status to stress adaptation [24,27]. It is notable that transgenic plants overexpressing *AKIN10* or *FUS3* gene are sensitive to abscisic acid (ABA), a pivotal stress hormone that modulates a broad spectrum of stress responses [45], and exhibit delayed seed germination [26]. SnRK1 kinases have also been implicated in aging process and cell death in eukaryotes [27,28], indicating that SnRK1 is a central regulator of sugar metabolism in linking plant development with environmental adaptation.

The observed role of IDD8 in the SnRK1-mediated control of photoperiodic flowering under sugar starvation conditions suggest that IDD8 function is not limited to flowering time control but might be extended to a range of stress responses. It has been observed that transgenic plants overexpressing *IDD8* gene exhibit a plethora of growth and developmental defects, such as growth retardation and architecturally distorted, pale-green leaves [20]. It will be worthy of examining the responses of *IDD8*-overexpressing plants and *idd8-3* mutant to ABA and abiotic stresses and investigating whether SnRK1 is involved in the IDD8-mediated stress responses.

## Conclusions

We aimed to improve our understanding on how IDD8 perceives sugar deprivation signals in regulating photoperiodic flowering. We found that the energy metabolic sensor SnRK1 inhibits the transcriptional activation activity of IDD8 transcription factor, which regulates photoperiodic flowering in response to sugar deprivation. AKIN10, the α-catalytic subunit of SnRK1 kinases, phosphorylates IDD8 predominantly at two serine residues, Ser-178 and Ser-182 that reside in the fourth ZF domain. While protein phosphorylation does not affect the nuclear localization and DNA-binding property of IDD8, it significantly reduces the transcriptional activation activity of IDD8. The reduction of the IDD8 activity was also observed under sugar starvation conditions, which is consistent with the activation of SnRK1 activity by low energy status. Our data show that the SnRK1-IDD8 transcriptional regulatory module serves as a web that integrates sugar metabolic signals into flowering time control in *Arabidopsis*.

## Methods

### Bioinformatics software

Nucleotide sequences of genes and amino acid sequences of proteins were obtained from the *Arabidopsis* Information Resource (TAIR, http://www.arabidopsis.org/). Protein phosphorylation sites were predicted using the NetPhos 2.0 software (http://www.cbs.dtu.dk/services/NetPhos/).

### Plant materials and growth conditions

All *Arabidopsis thaliana* lines used were in the Col-0 background. *Arabidopsis* plants were grown in a controlled culture room set at 23°C with relative humidity of 55% under LDs with white light illumination (120 μM photons m^−2^s^−1^) provided by fluorescent FLR40D/A tubes (Osram, Seoul, Korea). The *idd8-3*, *akin10-1*, and *akin11-1* mutants have been described previously [20,26,45].

To generate 35S:*MYC*-*IDD8* transgenic plant, a full-size *IDD8* cDNA (At5g44160) was fused in-frame to the 3^′^ end of the MYC-coding sequence under the control of the CaMV 35S promoter in the myc-pBA vector [46]. The expression construct was transformed into Col-0 plants. To generate transgenic plants overexpressing *AKIN10* and *AKIN11* genes (At3g01090 and At3g29160, respectively), full-size cDNAs were subcloned under the control of the CaMV 35S promoter into the binary pB2GW7 vector [47]. *Agrobacterium*-mediated transformation was performed according to a modified floral-dip method [48].

For dark treatments, 10-day-old plants grown on ^1^/_2_ X Murashige and Skoog-agar plates (MS-agar plates) were covered with aluminum foil and incubated at 23°C for 2 days in complete darkness. For DCMU treatments, 10-day-old plants grown on MS-agar plates were transfer to MS liquid culture containing 50 μM DCMU for 2 days under constant light conditions.

### Gene expression analysis

Gene transcript levels were analyzed by qRT-PCR. Reverse transcription and quantitative PCR reaction were performed according to the rules that have been proposed to ensure reproducible and accurate measurements of transcript levels [49]. Total RNA samples were pretreated with RNase-free DNase to get rid of any contaminating genomic DNA before use.

qRT-PCR reactions were performed in 96-well blocks with the Applied Biosystems 7500 Real-Time PCR System (Foster City, CA) using the SYBR Green I master mix in a volume of 20 μl. The PCR primers were designed using the Primer Express Software installed into the system and listed in Additional file 11. The two-step thermal cycling profile used was at 94°C for 15 s and at 68°C for 1 min. An *eIF4A* gene (At3g13920) was included in the reactions as internal control for normalizing the variations in the cDNA amounts used. All qRT-PCR reactions were performed in biological triplicates using RNA samples extracted from three independent plant materials grown under identical conditions. The comparative ΔΔC_T_ method was employed to evaluate the relative quantity of each amplified product in the samples. The threshold cycle (C_T_) was automatically determined for each reaction by the system set with default parameters. The specificity of the PCR reactions was determined by melting curve analysis of the amplified products using the standard method installed in the system.

### Flowering time measurement

Plants were grown in soil at 23°C under LDs until flowering. Flowering times were determined by counting the days to bolting and the number of rosette and cauline leaves at bolting. Fifteen to 20 plants were counted and averaged for each measurement.

### *in vitro* pull-down assay

Recombinant AKIN10 and AKIN11 proteins were prepared as GST-AKIN10 and GST-AKIN11 fusions in *Escherichia coli* Rosetta2 (DE3) pLysS strain (Novagen, Madison, WI) and affinity-purified, as described previously [50]. The [^35^S] methionine-labeled IDD8 polypeptides were prepared by *in vitro* translation using the TNT-coupled reticulocyte lysate system (Promega, Madison, WI).

The *in vitro* pull-down assays were performed as described previously [50]. The bound proteins were eluted with 1X SDS-PAGE loading buffer by boiling for 5 min and subjected to SDS- PAGE and autoradiography.

### Subcellular localization assay

A GFP-coding sequence was fused in-frame to the 5′ end of *IDD8* gene, and the gene fusion was subcloned into the p2FGW7 expression vector (Invitrogen, Carlsbad, CA). Protoplasts were prepared from fully expanded leaves of four-week-old plants grown in soil, as described previously [51]. Approximately 2 × 10^4^ protoplasts were mixed with 10 μg of plasmid DNA and 110 μl of polyethylene glycol (PEG)-calcium transfection solution [40% PEG 4000 (w/v), 0.2 M mannitol, 100 mM CaCl_2_]. After incubation at 22°C for 15 min, the protoplast suspension was centrifuged at 100 × g for 2 min. The protoplasts were resuspended in 1 ml of WI solution (0.5M mannitol, 4 mM Mes, pH 5.7, 20 μΜ KCl) and incubated in the dark at 22°C for 15 h. The subcellular distribution of green fluorescence was visualized by fluorescence microscopy.

The *GFP-IDD8* gene fusion was overexpressed under the control of the CaMV 35S promoter in Col-0 and *10*-ox plants. The roots of ten-day-old transgenic plants were visualized by differential interference contrast (DIC) and fluorescence microscopy. The root samples were also stained with 4′,6-diamidino-2-phenylindole (DAPI) to visualize the nuclei.

### Liquid chromatography-tandem mass spectrometry (LC-MS/MS)

Recombinant MBP-IDD8 and GST-AKIN10 protein fusions, in which the tags were fused to the N termini of the proteins, were prepared in *E. coli* cells. Phosphorylation reactions *in vitro* were induced by incubating with non-radioactive ATP. The MBP-IDD8 protein was excised from 6% SDS-PAGE gel and digested with trypsin. LC-MS/MS was performed in the National Instrumentation Center for Environmental Management (NICEM, Seoul National University, Seoul). Protein Pilot program (Applied Biosystems, Foster City, CA) was used to assign the phosphorylation sites. The serine (S) and threonine (T) phosphorylation sites identified by the Protein Pilot program were calculated with a confidence > 0.95.

### *in vitro* protein phosphorylation assay

The assays were performed in 10 μl kinase reaction buffer (20 mM HEPES, pH 7.4, 10 mM MgCl_2_, 1 mM Na_3_VO_4_, 2 mM DTT, 0.5 mM PMSF, 2 mM EDTA), as described previously [50]. Purified recombinant AKIN10 and IDD8 proteins were added to the reaction buffer supplemented with 1 μCi of [γ^−32^P] ATP. The reaction mixture was incubated at 30°C for 30 min, and the reaction was terminated by adding 4 μl of 6 X SDS-PAGE sample loading buffer. The mixture was boiled for 5 min before loading onto 10% SDS-PAGE gels. The gels were stained with Coomassie Brilliant Blue R250, vacuum-dried onto 3MM paper, and subjected to autoradiography.

### BiFC assay

BiFC assays were performed as described previously [51]. A full-size *IDD8* cDNA was fused in-frame to the 3′ end of the gene sequence encoding the N-terminal half of enhanced yellow fluorescent protein (EYFP) in the pSATN-nEYFP-C1 vector (E3081). A full-size *AKIN10* cDNA was fused in-frame to the 5′ end of the gene sequence encoding the C-terminal half of EYFP in the pSATN-cEYFP-C1 vector (E3082). The nYFP-IDD8 and AKIN10-cYFP vectors were cotransfected into *Arabidopsis* mesophyll protoplasts by the PEG-calcium transfection method [51]. The transfected protoplasts were incubated at 23°C for 16 h. The subcellular localization of IDD8-AKIN10 complexes was monitored by DIC microscopy and fluorescence microscopy. Reconstitution of YFP fluorescence was observed using a Zeiss LSM510 confocal microscope (Carl Zeiss, Yena, Germany) with the following YFP filter set up: excitation 515 nm, 458/514 dichroic, and emission 560- to 615-nm band-pass filter.

### Chromatin immunoprecipitation (ChIP) assay

ChIP assays were performed using two-week-old plants grown on MS-agar plates, as described previously [52]. Whole plants were vacuum-infiltrated with 1% (v/v) formaldehyde for cross-linking and ground in liquid nitrogen after quenching the cross-linking process. Chromatin preparations were sonicated into 0.4- to 0.7-kb fragments and precleared with salmon sperm DNA/Protein G agarose beads (Roche, Indianapolis, IN), and an anti-MYC antibody (Millipore, Billerica, MA) was added to the mixture. The precipitates were eluted from the beads, and cross-links were reversed. Residual proteins were removed by incubation with proteinase K. DNA was then recovered using a SV minicolumn (Promega). Quantitative PCR was performed to determine the amounts of genomic DNA enriched in the chromatin preparations.

### Transcriptional activation activity assay

For transcriptional activation activity assays, a series of reporter and effector vectors was constructed. In the reporter vector, four copies of the GAL4 upstream activation sequence (UAS) were fused to a gene encoding GUS. A full-size *IDD8* cDNA was fused to the GAL4 DNA-binding domain-coding sequence under the control of the CaMV 35S promoter in the effector vector. Full-size *AKIN10* and *AKIN11* cDNAs were subcloned into the expression vector harboring the CaMV 35S promoter. A positive control was ARF5M construct, in which a full-size *ARF5M* cDNA was subcloned into the GAL4 expression vector [53]. The reporter, effector, and expression vectors were cotransformed into *Arabidopsis* mesophyll protoplasts by the PEG-calcium transfection method [51]. The CaMV 35S promoter-luciferase construct was also cotransformed as an internal control. GUS activity was measured by the fluorometric method as described previously [54]. Luciferase activity assays were performed using the Luciferase Assay System kit (Promega).

### Accession numbers

The *Arabidopsis* Genome Initiative locus identifiers for the genes mentioned in this article are: At5g44160, *IDD8*; At3g01090, *AKIN10*; At3g29160, *AKIN11*; At3g43190, *SUS4*; At3g13920, *eIF4A*; At1g22710, *SUC2*; At5g43610, *SUC6*; At1g66570, *SUC7*; At2g14670, *SUC8*; and At3g26744, *ICE1*.

### Availability of supporting data

The data sets supporting the results of this article are included within the article and its additional files.

## Additional files

Additional file 1:
**Molecular characterization of** ***akin10-1*** **and** ***akin11-1*** **mutants.** **A**. Mapping of T-DNA insertions. The *AKIN10*-defective *akin10-1* (SALK-127939) and *AKIN11*-defective *akin11-1* (WiscDsLox320B03) mutants were isolated from a pool of T-DNA insertional lines deposited in the *Arabidopsis* Biological Resource Center (ABRC, Ohio State University, OH). Black boxes represent exons, and white boxes represent 5′ and 3′ untranslated regions. F and R, forward and reverse primers, respectively. bp, base pair. **B**. *AKIN10* expression in *akin10-1* mutant. Gene expression was examined by PCR-based genotyping (left panel), in which left and right primers (LP and RP, respectively) that are specific to the flanking sequences of the T-DNA insertion site and a T-DNA-specific LBb1.3 primer were used, quantitative real-time RT-PCR (qRT-PCR, middle panel), and RT-PCR (right panel). The primer sequences were obtained from the Salk Institute Genomic Analysis Laboratory (http://signal.salk.edu/cgi-bin/tdnaexpress). SM, size marker. In RT-PCR, a tubulin gene (*TUB*) was included as control of constitutive expression. In qRT-PCR, biological triplicates were averaged and statistically treated using Student *t*-test (**P* < 0.01, difference from Col-0). Bars indicate standard error of the mean. **C**. *AKIN11* expression in *akin11-1* mutant. Gene expression was examined by PCR-based genotyping (left panel) and qRT-PCR (right panel), as described in (**B**). The T-DNA-specific P745 primer sequence was obtained from the *Arabidopsis* Information Resource (TAIR, http://www.arabidopsis.org/). Bars indicate standard error of the mean (*t*-test,**P* < 0.01, difference from Col-0). Additional file 2:
**Expression of transgenes in** ***10*** **-ox and** ***11-*** **ox transgenic plants.** Four independent transgenic plants overexpressing *AKIN10* (*10*-ox) and *AKIN11* (*11-*ox) genes were grown on ½ X Murashige and Skoog-agar plates (hereafter, referred to as MS-agar plates) for 2 weeks under long days (LDs, 16-h light and 8-h dark) before harvesting whole plant materials for total RNA extraction. Transcript levels of *AKIN10* gene (**A**) and *AKIN11* gene (**B**) were determined by qRT-PCR. Biological triplicates were averaged and statistically analyzed using Student *t*-test (**P* < 0.01, difference from Col-0). Bars indicate standard error of the mean. Additional file 3:
**Expression of IDD8 downstream genes in** ***10*** **-ox plant and** ***akin10-1*** **mutant.** *AKIN10*-overexpressing (*10*-ox) and -deficient (*akin10-1*) plants were grown on MS-agar plates for 2 weeks under LDs before harvesting whole plant materials for total RNA extraction. Transcript levels of *SUCROSE SYNTHASE 4* (*SUS4*) gene (**A**), which is a target of IDD8, and *SUCROSE-PROTON SYMPORTER* (*SUC*) genes (**B**), which function downstream of IDD8, were determined by qRT-PCR. Biological triplicates were averaged and statistically analyzed using Student *t*-test (**P* < 0.01, difference from Col-0). Bars indicate standard error of the mean. Additional file 4:
**Bimolecular fluorescence complementation (BiFC) assay** ***.*** The nYFP-IDD8 and cYFP-AKIN fusions were coexpressed with cYFP vector and nYFP vector, respectively, in *Arabidopsis* protoplasts. A cyan fluorescent protein (CFP)-tagged ICE1 nuclear marker was also coexpressed in *Arabidopsis* protoplasts. The protoplasts were visualized by differential interference contrast microscopy (DIC) and fluorescence microscopy. Scale bars, 10 μm. Additional file 5:
**Immunological detection of IDD8 and AKIN proteins** ***.*** Recombinant GST-AKIN and MBP-IDD8 proteins used in phosphorylation assays *in vitro* were detected immunologically using anti-GST and anti-MBP antibodies. Wild-type and mutated IDD8 proteins, which were used in Figures 3 and 4, were detected in (**A**) and (**B**), respectively. S, serine. T, threonine. A, alanine. kDa, kilodalton. Additional file 6:
**Amino acid sequences surrounding T98, S178, and S182 in IDD8 protein.** The consensus sequence for the SnRK1-mediated phosphorylation of serine (S) and threonine (T) residues is shown (upper panel). Amino acid sequences surrounding the putative phosphorylation residues of IDD8, such as T98, S178, and S182, are shown in the lower panel. The phosphorylated S and T residues are marked in bold and underline. Basic residues are marked in black shadow, and hydrophobic residues are marked in grey shadow. Additional file 7:
**Analysis of phosphorylated residues in IDD8 protein by mass spectrometry.** Recombinant MBP-IDD8 and GST-AKIN10 protein fusions were prepared in *E. coli* cells. Phosphorylation reactions *in vitro* were induced by incubating with non-radioactive ATP. MBP-IDD8 protein was excised from 6% SDS-PAGE gel, digested with trypsin, and analyzed by liquid chromatography-tandem mass spectrometry (LC-MS/MS). Protein Pilot program (Applied Biosystems, Foster City, CA) was used to assign the phosphorylation sites. The serine (S) and threonine (T) phosphorylation sites identified by the Protein Pilot program were calculated with a confidence > 0.95. Additional file 8:
**Effects of AKIN10 on protein stability and nuclear localization of IDD8.** **A**. IDD8 protein stability. The *IDD8-MYC* fusion was overexpressed driven by the Cauliflower Mosaic Virus (CaMV) 35S promoter in either Col-0 plant or *akin10-1* mutant. Ten-day-old plants grown on MS-agar plates were incubated for 2 days either in complete darkness or in the light in the presence or absence (mock) of 50 μM DCMU, a specific inhibitor of photosynthesis. Protein extracts were prepared from whole plant materials. IDD8 proteins were detected immunologically using an anti-MYC antibody (upper panel). Part of Coomassie Blue-stained gel was displayed as a loading control (middle panel). Total RNA was extracted from the light-grown plants, and transcript levels of *IDD8* gene were determined by qRT-PCR (lower left panel). Biological triplicates were averaged and statistically analyzed (*t*-test, **P* < 0.01, difference from Col-0 background). Relative protein intensity was calculated by dividing the band intensity with the transcript level (lower right panel). Bars indicate standard error of the mean. **B**. Subcellular localization of IDD8 in *Arabidopsis* protoplasts. A green fluorescent protein (GFP)-coding sequence was fused in-frame to the 5′ end of a full-size *IDD8* cDNA. The *GFP-IDD8* fusion was transiently expressed in *Arabidopsis* protoplasts and visualized by fluorescence microscopy. Chloroplasts appear red because of autofluorescence. Scale bars, 10 μm. **C**. Subcellular localization of IDD8 in the roots of 35S:*GFP*-*IDD8* transgenic plants. The transgenic were generated from Col-0 and *10-*ox plants. Roots of ten-day-old plants grown on MS-agar plates were visualized by DIC and fluorescence microscopy. The roots were also stained with 4′,6-diamidino-2-phenylindole (DAPI) to visualize the nuclei. Scale bars, 10 μm. Additional file 9:
**IDD8 binding to** ***SUS4*** **promoter in** ***akin10-1*** **mutant.** **A**. IDD8-binding sequence in *SUS4* promoter. The IDD8-binding sequence (IDD8-BS) containing a conserved CTTTTGTCC motif covers residues −2553 to −2348 upstream of the translation start site. A non-binding sequence (IDD8-nBS) covering residues −1363 to −1158 was included as negative control in the assay. Black boxes indicate exons, and white boxes indicate 5′ and 3′ untranslated regions. kbp, kilobase pair. **B**. Chromatin immunoprecipitation (ChIP) assay on IDD8 binding to *SUS4* chromatin. Plants grown on MS-agar plates for 12 days under LDs were used for chromatin preparation. An *eIF4A* DNA fragment was used for normalization. Four measurements were averaged for each plant genotype and statistically analyzed using Student *t*-test (**P* < 0.01, difference from mock). Bars indicate standard error of the mean. IP, immunoprecipitation. Additional file 10:
**Expression of** ***IDD8*** **and** ***AKIN10*** **genes in** ***idd8-3 akin10-1*** **double mutant.** Ten-day-old plants grown on MS-agar plates were used for total RNA extraction. Transcript levels were determined by qRT-PCR. Biological triplicates were averaged and statistically analyzed using Student *t*-test (**P* < 0.01, difference from Col-0). Bars indicate standard error of the mean. Additional file 11:
**Primers used.** F, forward primer; R, reverse primer.

## Abbreviations

IDD: INDETERMINATE DOMAIN
SNF1: Sucrose non-fermenting 1
SnRK1: SNF1-related protein kinase 1
AKIN10: *Arabidopsis* SNF1 kinase homolog 10
ZF: Zinc finger
GA: Gibberellic acid
FT: FLOWERING LOCUS T
SOC1: SUPPRESSOR OF CONSTANS OVEREXPRESSION 1
FLC: FLOWERING LOCUS C
AtSUC9: SUCROSE TRANSPORTER9
T6P: Trehalose-6-phosphate
CO: CONSTANS
SUS: Sucrose synthase
AMPK1: AMP-dependent protein kinase 1
CaMV: Cauliflower mosaic virus
GST: Glutathione S-transferase
BiFC: Bimolecular fluorescence complementation
YFP: Yellow fluorescence protein
MBP: Maltose-binding protein
FUS3: FUSCA3
DCMU: 3-(3,4-dichlorophenyl)-1,1-dimethylurea
qRT-PCR: Quantitative real-time RT-PCR
GFP: Green fluorescence protein
ChIP: Chromatin immunoprecipitation
GUS: β-glucuronidase
AP1: APPETALA 1
LD: Long day
LC-MS/MS: Liquid chromatography-tandem mass spectrometry
