# Supplementary material for: AKIN10 delays flowering by inactivating IDD8 transcription factor through protein phosphorylation in Arabidopsis
Source: BMC Plant Biol. 2015 May 1;15:110. doi: 10.1186/s12870-015-0503-8 (PMC4416337; doi:10.1186/s12870-015-0503-8)
Supplement: Additional file 1: — Molecular characterization of akin10-1 and akin11-1 mutants. A. Mapping of T-DNA insertions. The AKIN10-defective akin10-1 (SALK-127939) and AKIN11-defective akin11-1 (WiscDsLox320B03) mutants were isolated from a pool of T-DNA insertional lines deposited in the Arabidopsis Biological Resource Center (ABRC, Ohio State University, OH). Black boxes represent exons, and white boxes represent 5′ and 3′ untranslated regions. F and R, forward and reverse primers, respectively. bp, base pair. B. AKIN10 expression in akin10-1 mutant. Gene expression was examined by PCR-based genotyping (left panel), in which left and right primers (LP and RP, respectively) that are specific to the flanking sequences of the T-DNA insertion site and a T-DNA-specific LBb1.3 primer were used, quantitative real-time RT-PCR (qRT-PCR, middle panel), and RT-PCR (right panel). The primer sequences were obtained from the Salk Institute Genomic Analysis Laboratory (http://signal.salk.edu/cgi-bin/tdnaexpress). SM, size marker. In RT-PCR, a tubulin gene (TUB) was included as control of constitutive expression. In qRT-PCR, biological triplicates were averaged and statistically treated using Student t-test (*P < 0.01, difference from Col-0). Bars indicate standard error of the mean. C. AKIN11 expression in akin11-1 mutant. Gene expression was examined by PCR-based genotyping (left panel) and qRT-PCR (right panel), as described in (B). The T-DNA-specific P745 primer sequence was obtained from the Arabidopsis Information Resource (TAIR, http://www.arabidopsis.org/). Bars indicate standard error of the mean (t-test,*P < 0.01, difference from Col-0). [file 12870_2015_503_MOESM1_ESM.pdf]

Additional file 1

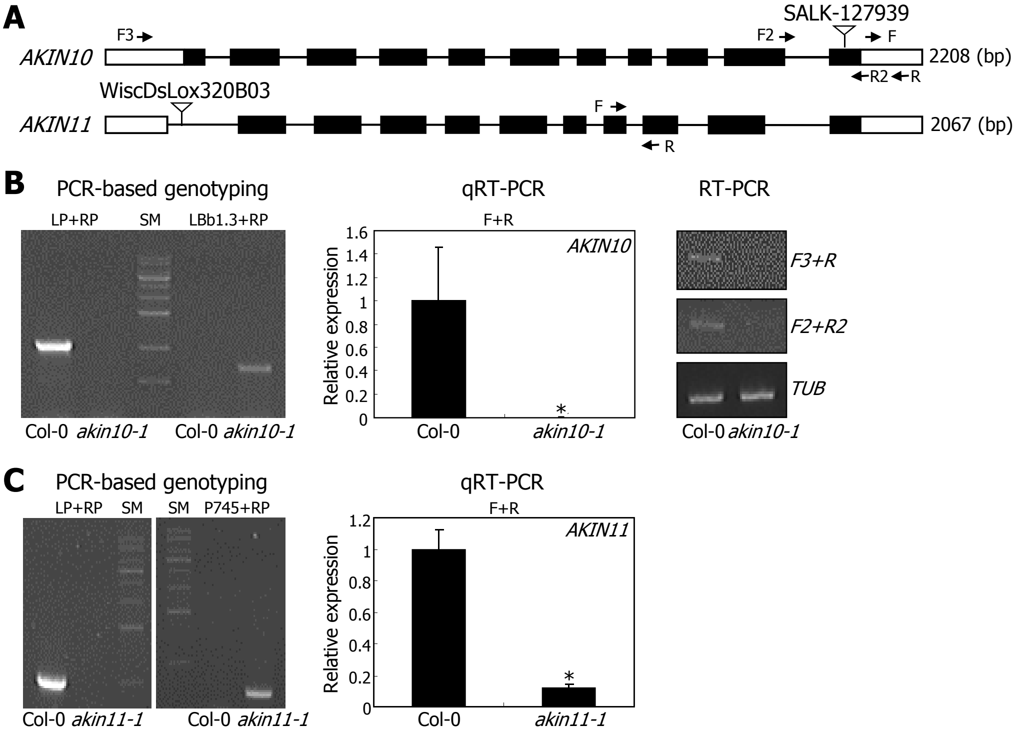

Additional file 1. Molecular characterization of *akin10-1* and *akin11-1* mutants.

A. Mapping of T-DNA insertions. The *AKIN10*-defective *akin10-1* (SALK-127939) and *AKIN11*-defective *akin11-1* (WiscDsLox320B03) mutants were isolated from a pool of T-DNA insertional lines deposited in the *Arabidopsis* Biological Resource Center (ABRC, Ohio State University, OH). Black boxes represent exons, and white boxes represent 5' and 3' untranslated regions. F and R, forward and reverse primers, respectively. bp, base pair.

B. *AKIN10* expression in *akin10-1* mutant. Gene expression was examined by PCR-based genotyping (left panel), in which left and right primers (LP and RP, respectively) that are specific to the flanking sequences of the T-DNA insertion site and a T-DNA-specific LBb1.3 primer were used, quantitative real-time RT-PCR (qRT-PCR, middle panel), and RT-PCR (right panel). The primer sequences were obtained from the Salk Institute Genomic Analysis Laboratory (http://signal.salk.edu/cgi-bin/tdnaexpress). SM, size marker. In RT-PCR, a tubulin gene (*TUB*) was included as control of constitutive expression. In qRT-PCR, biological triplicates were averaged and statistically treated using Student *t*-test (*$P < 0.01$, difference from Col-0). Bars indicate standard error of the mean.

C. *AKIN11* expression in *akin11-1* mutant. Gene expression was examined by PCR-based genotyping (left panel) and qRT-PCR (right panel), as described in (**B**). The T-DNA-specific P745 primer sequence was obtained from the *Arabidopsis* Information Resource (TAIR, http://www.arabidopsis.org/). Bars indicate standard error of the mean (*t*-test,*$P < 0.01$, difference from Col-0).
