# Supplementary material for: AKIN10 delays flowering by inactivating IDD8 transcription factor through protein phosphorylation in Arabidopsis
Source: BMC Plant Biol. 2015 May 1;15:110. doi: 10.1186/s12870-015-0503-8 (PMC4416337; doi:10.1186/s12870-015-0503-8)
Supplement: Additional file 2: — Expression of transgenes in 10 -ox and 11- ox transgenic plants. Four independent transgenic plants overexpressing AKIN10 (10-ox) and AKIN11 (11-ox) genes were grown on ½ X Murashige and Skoog-agar plates (hereafter, referred to as MS-agar plates) for 2 weeks under long days (LDs, 16-h light and 8-h dark) before harvesting whole plant materials for total RNA extraction. Transcript levels of AKIN10 gene (A) and AKIN11 gene (B) were determined by qRT-PCR. Biological triplicates were averaged and statistically analyzed using Student t-test (*P < 0.01, difference from Col-0). Bars indicate standard error of the mean. [file 12870_2015_503_MOESM2_ESM.pdf]

Additional file 2

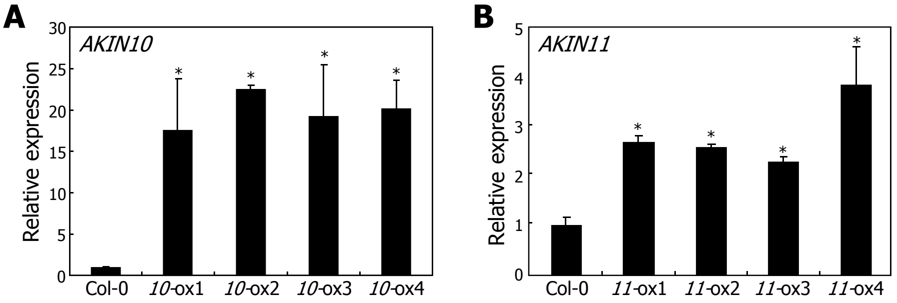

Additional file 2. Expression of transgenes in *10*-ox and *11*-ox transgenic plants.

Four independent transgenic plants overexpressing *AKIN10* (*10*-ox) and *AKIN11* (*11*-ox) genes were grown on ½ X Murashige and Skoog-agar plates (hereafter, referred to as MS-agar plates) for 2 weeks under long days (LDs, 16-h light and 8-h dark) before harvesting whole plant materials for total RNA extraction. Transcript levels of *AKIN10* gene (**A**) and *AKIN11* gene (**B**) were determined by qRT-PCR. Biological triplicates were averaged and statistically analyzed using Student *t*-test (**P* < 0.01, difference from Col-0). Bars indicate standard error of the mean.
