# Supplementary material for: AKIN10 delays flowering by inactivating IDD8 transcription factor through protein phosphorylation in Arabidopsis
Source: BMC Plant Biol. 2015 May 1;15:110. doi: 10.1186/s12870-015-0503-8 (PMC4416337; doi:10.1186/s12870-015-0503-8)
Supplement: Additional file 3: — Expression of IDD8 downstream genes in 10 -ox plant and akin10-1 mutant. AKIN10-overexpressing (10-ox) and -deficient (akin10-1) plants were grown on MS-agar plates for 2 weeks under LDs before harvesting whole plant materials for total RNA extraction. Transcript levels of SUCROSE SYNTHASE 4 (SUS4) gene (A), which is a target of IDD8, and SUCROSE-PROTON SYMPORTER (SUC) genes (B), which function downstream of IDD8, were determined by qRT-PCR. Biological triplicates were averaged and statistically analyzed using Student t-test (*P < 0.01, difference from Col-0). Bars indicate standard error of the mean. [file 12870_2015_503_MOESM3_ESM.pdf]

Additional file 3

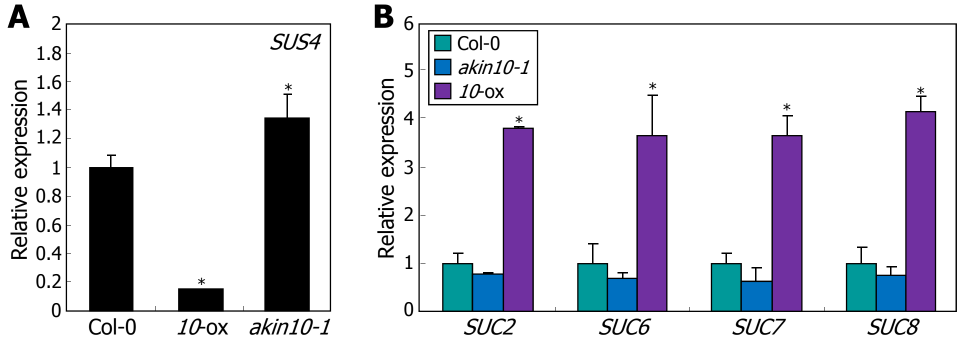

Additional file 3. Expression of IDD8 downstream genes in *10*-ox plant and *akin10-1* mutant.

AKIN10-overexpressing (*10*-ox) and -deficient (*akin10-1*) plants were grown on MS-agar plates for 2 weeks under LDs before harvesting whole plant materials for total RNA extraction. Transcript levels of *SUCROSE SYNTHASE 4* (*SUS4*) gene (**A**), which is a target of IDD8, and *SUCROSE-PROTON SYMPORTER* (*SUC*) genes (**B**), which function downstream of IDD8, were determined by qRT-PCR. Biological triplicates were averaged and statistically analyzed using Student *t*-test (*$P < 0.01$, difference from Col-0). Bars indicate standard error of the mean.
