# Supplementary material for: AKIN10 delays flowering by inactivating IDD8 transcription factor through protein phosphorylation in Arabidopsis
Source: BMC Plant Biol. 2015 May 1;15:110. doi: 10.1186/s12870-015-0503-8 (PMC4416337; doi:10.1186/s12870-015-0503-8)
Supplement: Additional file 4: — Bimolecular fluorescence complementation (BiFC) assay . The nYFP-IDD8 and cYFP-AKIN fusions were coexpressed with cYFP vector and nYFP vector, respectively, in Arabidopsis protoplasts. A cyan fluorescent protein (CFP)-tagged ICE1 nuclear marker was also coexpressed in Arabidopsis protoplasts. The protoplasts were visualized by differential interference contrast microscopy (DIC) and fluorescence microscopy. Scale bars, 10 μm. [file 12870_2015_503_MOESM4_ESM.pdf]

Additional file 4

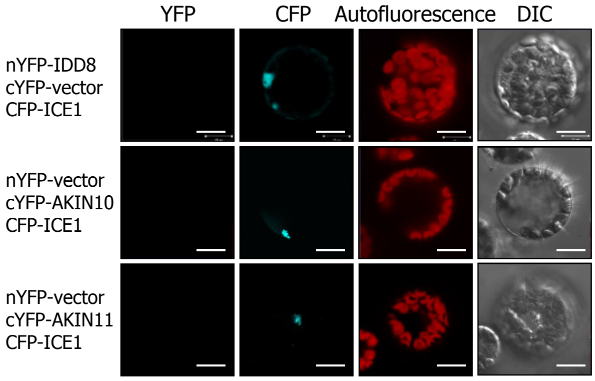

Additional file 4. Bimolecular fluorescence complementation (BiFC) assay.

The nYFP-IDD8 and cYFP-AKIN fusions were coexpressed with cYFP vector and nYFP vector, respectively, in *Arabidopsis* protoplasts. A cyan fluorescent protein (CFP)-tagged ICE1 nuclear marker was also coexpressed in *Arabidopsis* protoplasts. The protoplasts were visualized by differential interference contrast microscopy (DIC) and fluorescence microscopy. Scale bars, 10 μm.
