# Supplementary material for: AKIN10 delays flowering by inactivating IDD8 transcription factor through protein phosphorylation in Arabidopsis
Source: BMC Plant Biol. 2015 May 1;15:110. doi: 10.1186/s12870-015-0503-8 (PMC4416337; doi:10.1186/s12870-015-0503-8)
Supplement: Additional file 5: — Immunological detection of IDD8 and AKIN proteins . Recombinant GST-AKIN and MBP-IDD8 proteins used in phosphorylation assays in vitro were detected immunologically using anti-GST and anti-MBP antibodies. Wild-type and mutated IDD8 proteins, which were used in Figures 3 and 4, were detected in (A) and (B), respectively. S, serine. T, threonine. A, alanine. kDa, kilodalton. [file 12870_2015_503_MOESM5_ESM.pdf]

Additional file 5

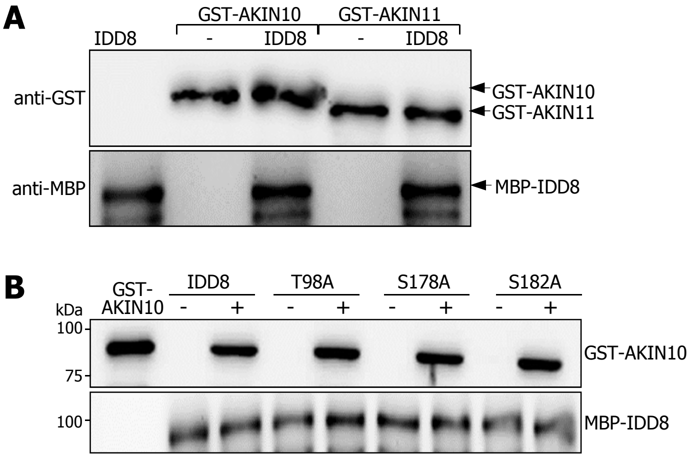

Additional file 5. Immunological detection of IDD8 and AKIN proteins.

Recombinant GST-AKIN and MBP-IDD8 proteins used in phosphorylation assays *in vitro* were detected immunologically using anti-GST and anti-MBP antibodies. Wild-type and mutated IDD8 proteins, which were used in Figures 3 and 4, were detected in (**A**) and (**B**), respectively. S, serine. T, threonine. A, alanine. kDa, kilodalton.
