# Supplementary material for: AKIN10 delays flowering by inactivating IDD8 transcription factor through protein phosphorylation in Arabidopsis
Source: BMC Plant Biol. 2015 May 1;15:110. doi: 10.1186/s12870-015-0503-8 (PMC4416337; doi:10.1186/s12870-015-0503-8)
Supplement: Additional file 6: — Amino acid sequences surrounding T98, S178, and S182 in IDD8 protein. The consensus sequence for the SnRK1-mediated phosphorylation of serine (S) and threonine (T) residues is shown (upper panel). Amino acid sequences surrounding the putative phosphorylation residues of IDD8, such as T98, S178, and S182, are shown in the lower panel. The phosphorylated S and T residues are marked in bold and underline. Basic residues are marked in black shadow, and hydrophobic residues are marked in grey shadow. [file 12870_2015_503_MOESM6_ESM.pdf]

Additional file 6

-5	-4	-3	-2	-1	0	1	2	3	4
M	X	R	X	X	**S**	X	X	X	L
L		K			**T**				F
V		H							I
F	R								M
I									V

T98	L	P	W	K	L	K	Q	R	**T**	S	K	E	V	I
S178	R	C	D	C	G	T	I	F	**S**	R	R	D	S	F
S182	G	T	I	F	S	R	R	D	**S**	F	I	T	H	R

Additional file 6. Amino acid sequences surrounding T98, S178, and S182 in IDD8 protein.

The consensus sequence for the SnRK1-mediated phosphorylation of S and T residues is shown (upper panel). Amino acid sequences surrounding the putative phosphorylation residues of IDD8, such as T98, S178, and S182, are shown in the lower panel. The phosphorylated S and T residues are marked in bold and underline. Basic residues are marked in black shadow, and hydrophobic residues are marked in grey shadow.
