# Supplementary material for: AKIN10 delays flowering by inactivating IDD8 transcription factor through protein phosphorylation in Arabidopsis
Source: BMC Plant Biol. 2015 May 1;15:110. doi: 10.1186/s12870-015-0503-8 (PMC4416337; doi:10.1186/s12870-015-0503-8)
Supplement: Additional file 7: — Analysis of phosphorylated residues in IDD8 protein by mass spectrometry. Recombinant MBP-IDD8 and GST-AKIN10 protein fusions were prepared in E. coli cells. Phosphorylation reactions in vitro were induced by incubating with non-radioactive ATP. MBP-IDD8 protein was excised from 6% SDS-PAGE gel, digested with trypsin, and analyzed by liquid chromatography-tandem mass spectrometry (LC-MS/MS). Protein Pilot program (Applied Biosystems, Foster City, CA) was used to assign the phosphorylation sites. The serine (S) and threonine (T) phosphorylation sites identified by the Protein Pilot program were calculated with a confidence > 0.95. [file 12870_2015_503_MOESM7_ESM.pdf]

Additional file 7

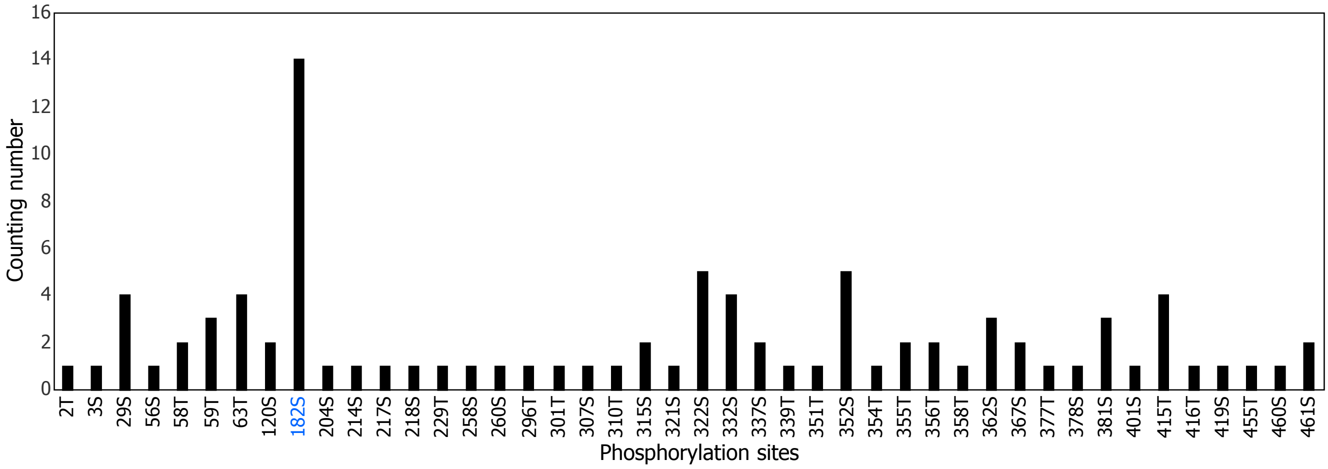

Additional file 7. Analysis of phosphorylated residues in IDD8 protein by mass spectrometry.

Recombinant MBP-IDD8 and GST-AKIN10 protein fusions were prepared in *E. coli* cells. Phosphorylation reactions *in vitro* were induced by incubating with non-radioactive ATP. MBP-IDD8 protein was excised from 6% SDS-PAGE gel, digested with trypsin, and analyzed by liquid chromatography-tandem mass spectrometry (LC-MS/MS). Protein Pilot program (Applied Biosystems, Foster City, CA) was used to assign the phosphorylation sites. The serine (S) and threonine (T) phosphorylation sites identified by the Protein Pilot program were calculated with a confidence > 0.95.
