# Supplementary material for: AKIN10 delays flowering by inactivating IDD8 transcription factor through protein phosphorylation in Arabidopsis
Source: BMC Plant Biol. 2015 May 1;15:110. doi: 10.1186/s12870-015-0503-8 (PMC4416337; doi:10.1186/s12870-015-0503-8)
Supplement: Additional file 8: — Effects of AKIN10 on protein stability and nuclear localization of IDD8. A. IDD8 protein stability. The IDD8-MYC fusion was overexpressed driven by the Cauliflower Mosaic Virus (CaMV) 35S promoter in either Col-0 plant or akin10-1 mutant. Ten-day-old plants grown on MS-agar plates were incubated for 2 days either in complete darkness or in the light in the presence or absence (mock) of 50 μM DCMU, a specific inhibitor of photosynthesis. Protein extracts were prepared from whole plant materials. IDD8 proteins were detected immunologically using an anti-MYC antibody (upper panel). Part of Coomassie Blue-stained gel was displayed as a loading control (middle panel). Total RNA was extracted from the light-grown plants, and transcript levels of IDD8 gene were determined by qRT-PCR (lower left panel). Biological triplicates were averaged and statistically analyzed (t-test, *P < 0.01, difference from Col-0 background). Relative protein intensity was calculated by dividing the band intensity with the transcript level (lower right panel). Bars indicate standard error of the mean. B. Subcellular localization of IDD8 in Arabidopsis protoplasts. A green fluorescent protein (GFP)-coding sequence was fused in-frame to the 5′ end of a full-size IDD8 cDNA. The GFP-IDD8 fusion was transiently expressed in Arabidopsis protoplasts and visualized by fluorescence microscopy. Chloroplasts appear red because of autofluorescence. Scale bars, 10 μm. C. Subcellular localization of IDD8 in the roots of 35S:GFP-IDD8 transgenic plants. The transgenic were generated from Col-0 and 10-ox plants. Roots of ten-day-old plants grown on MS-agar plates were visualized by DIC and fluorescence microscopy. The roots were also stained with 4′,6-diamidino-2-phenylindole (DAPI) to visualize the nuclei. Scale bars, 10 μm. [file 12870_2015_503_MOESM8_ESM.pdf]

Additional file 8

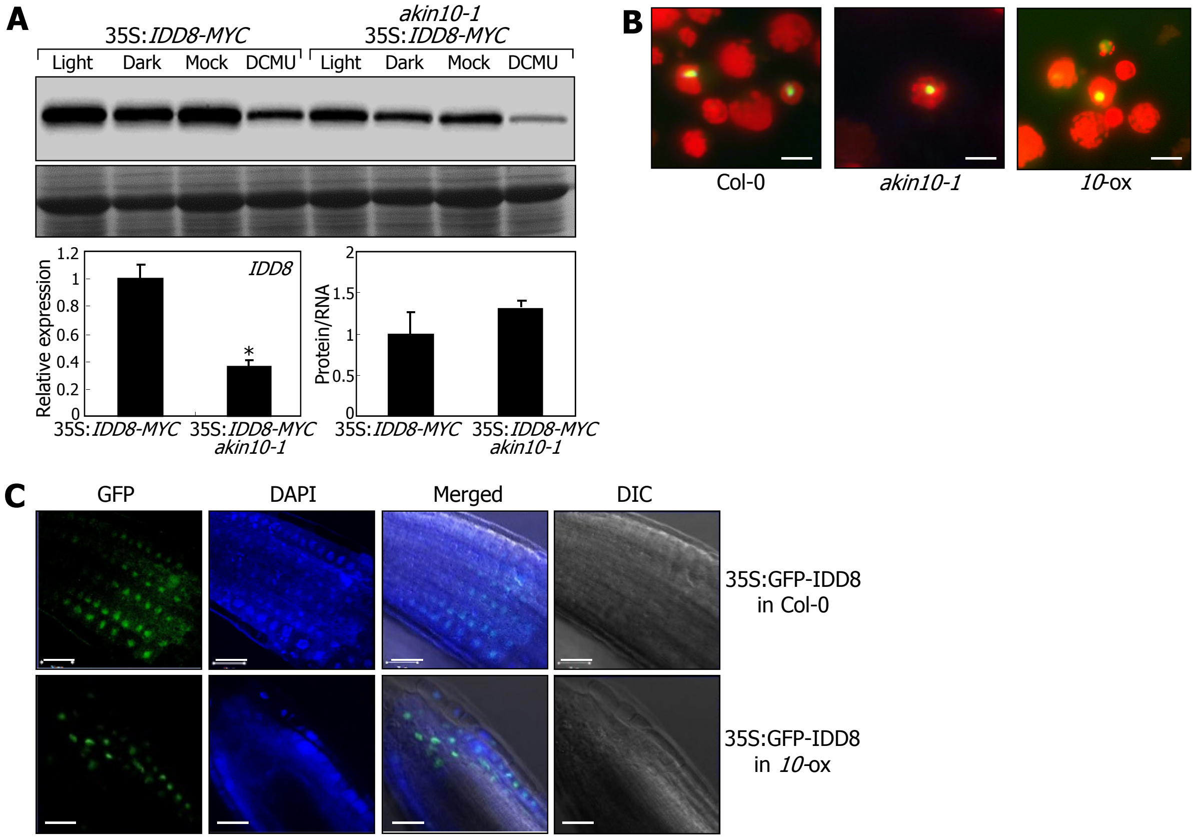

Additional file 8. Effects of AKIN10 on protein stability and nuclear localization of IDD8.

A. IDD8 protein stability. The *IDD8-MYC* fusion was overexpressed driven by the Cauliflower Mosaic Virus (CaMV) 35S promoter in either Col-0 plant or *akin10-1* mutant. Ten-day-old plants grown on MS-agar plates were incubated for 2 days either in complete darkness or in the light in the presence or absence (mock) of 50 µM DCMU, a specific inhibitor of photosynthesis. Protein extracts were prepared from whole plant materials. IDD8 proteins were detected immunologically using an anti-MYC antibody (upper panel). Part of Coomassie blue-stained gel was displayed as a loading control (middle panel). Total RNA was extracted from the light-grown plants, and transcript levels of *IDD8* gene were determined by qRT-PCR (lower left panel). Biological triplicates were averaged and statistically analyzed (*t*-test, $*P < 0.01$, difference from Col-0 background). Relative protein intensity was calculated by dividing the band intensity with the transcript level (lower right panel). Bars indicate standard error of the mean.

B. Subcellular localization of IDD8 in *Arabidopsis* protoplasts. A green fluorescent protein (GFP)-coding sequence was fused in-frame to the 5’ end of a full-size *IDD8* cDNA. The *GFP-IDD8* fusion was transiently expressed in *Arabidopsis* protoplasts and visualized by fluorescence microscopy. Chloroplasts appear red because of autofluorescence. Scale bars, 10 µm.

C. Subcellular localization of IDD8 in the roots of 35S:*GFP-IDD8* transgenic plants. The transgenic were generated from Col-0 and *10*-ox plants. Roots of ten-day-old plants grown on MS-agar plates were visualized by DIC and fluorescence microscopy. The roots were also stained with 4’,6-diamidino-2-phenylindole (DAPI) to visualize the nuclei. Scale bars, 10 µm.
