# Supplementary material for: AKIN10 delays flowering by inactivating IDD8 transcription factor through protein phosphorylation in Arabidopsis
Source: BMC Plant Biol. 2015 May 1;15:110. doi: 10.1186/s12870-015-0503-8 (PMC4416337; doi:10.1186/s12870-015-0503-8)
Supplement: Additional file 9: — IDD8 binding to SUS4 promoter in akin10-1 mutant. A. IDD8-binding sequence in SUS4 promoter. The IDD8-binding sequence (IDD8-BS) containing a conserved CTTTTGTCC motif covers residues −2553 to −2348 upstream of the translation start site. A non-binding sequence (IDD8-nBS) covering residues −1363 to −1158 was included as negative control in the assay. Black boxes indicate exons, and white boxes indicate 5′ and 3′ untranslated regions. kbp, kilobase pair. B. Chromatin immunoprecipitation (ChIP) assay on IDD8 binding to SUS4 chromatin. Plants grown on MS-agar plates for 12 days under LDs were used for chromatin preparation. An eIF4A DNA fragment was used for normalization. Four measurements were averaged for each plant genotype and statistically analyzed using Student t-test (*P < 0.01, difference from mock). Bars indicate standard error of the mean. IP, immunoprecipitation. [file 12870_2015_503_MOESM9_ESM.pdf]

Additional file 9

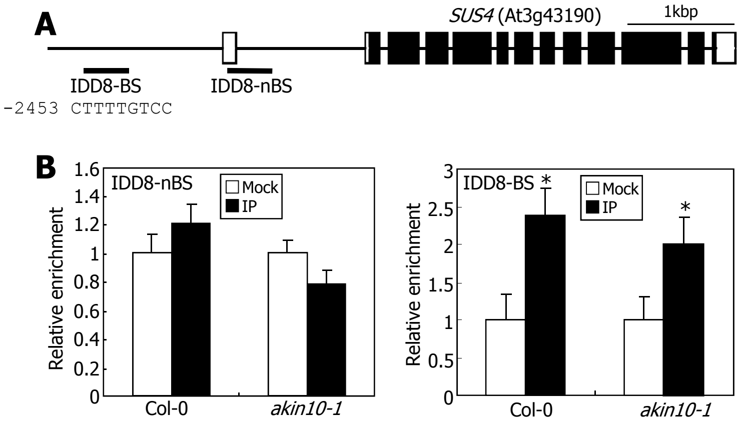

Additional file 9. IDD8 binding to *SUS4* promoter in *akin10-1* mutant.

A. IDD8-binding sequence in *SUS4* promoter. The IDD8-binding sequence (IDD8-BS) containing a conserved CTTTTGTCC motif covers residues -2553 to -2348 upstream of the translation start site. A non-binding sequence (IDD8-nBS) covering residues -1363 to -1158 was included as negative control in the assay. Black boxes indicate exons, and white boxes indicate 5' and 3' untranslated regions. kbp, kilobase pair.

B. Chromatin immunoprecipitation (ChIP) assay on IDD8 binding to *SUS4* chromatin. Plants grown on MS-agar plates for 12 days under LDs were used for chromatin preparation. An *eIF4A* DNA fragment was used for normalization. Four measurements were averaged for each plant genotype and statistically analyzed using Student *t*-test (**P* < 0.01, difference from mock). Bars indicate standard error of the mean. IP, immunoprecipitation.
