# Supplementary material for: AKIN10 delays flowering by inactivating IDD8 transcription factor through protein phosphorylation in Arabidopsis
Source: BMC Plant Biol. 2015 May 1;15:110. doi: 10.1186/s12870-015-0503-8 (PMC4416337; doi:10.1186/s12870-015-0503-8)
Supplement: Additional file 10: — Expression of IDD8 and AKIN10 genes in idd8-3 akin10-1 double mutant. Ten-day-old plants grown on MS-agar plates were used for total RNA extraction. Transcript levels were determined by qRT-PCR. Biological triplicates were averaged and statistically analyzed using Student t-test (*P < 0.01, difference from Col-0). Bars indicate standard error of the mean. [file 12870_2015_503_MOESM10_ESM.pdf]

Additional file 10

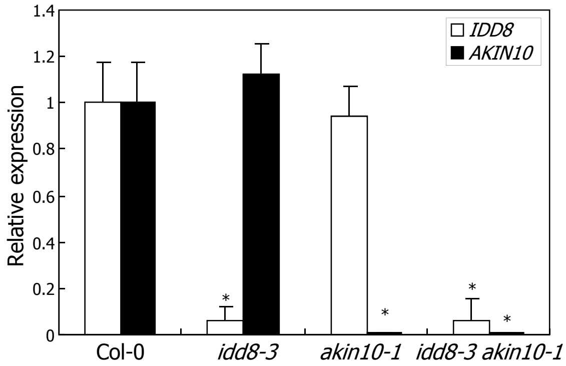

Additional file 10. Expression of *IDD8* and *AKIN10* genes in *idd8-3 akin10-1* double mutant.

Ten-day-old plants grown on MS-agar plates were used for total RNA extraction. Transcript levels were determined by qRT-PCR. Biological triplicates were averaged and statistically analyzed using Student *t*-test (**P* < 0.01, difference from Col-0). Bars indicate standard error of the mean.
