# Supplementary material for: AKIN10 delays flowering by inactivating IDD8 transcription factor through protein phosphorylation in Arabidopsis
Source: BMC Plant Biol. 2015 May 1;15:110. doi: 10.1186/s12870-015-0503-8 (PMC4416337; doi:10.1186/s12870-015-0503-8)
Supplement: Additional file 11: — Primers used. F, forward primer; R, reverse primer. [file 12870_2015_503_MOESM11_ESM.pdf]

Additional file 11

Primer	Usage	Sequence
eiF4A-F	qRT-PCR, RT-PCR	5′-TGACCACACAGTCTCTGCAA
eifF4A-R	qRT-PCR, RT-PCR	5′-ACCAGGGAGACTTGTTGGAC
ChIP-pSUS4-nBD-F	qRT-PCR, RT-PCR	5′-CTCCCTCCATCTTTCGCTTC
ChIP-pSUS4-nBD-R	qRT-PCR, RT-PCR	5′-TGCTACGGAAAGTCACTGGC
ChIP-pSUS4-BD-F	qRT-PCR, RT-PCR	5′-GAGCCGAAAACTCGTTGCAT
ChIP-pSUS4-BD-R	qRT-PCR, RT-PCR	5′-CGACCCAAATCAGGTCTTTG
AKIN10-F	qRT-PCR, RT-PCR	5'-TTTCTATATGCGTGCTCTACTCAAA
AKIN10-R	qRT-PCR, RT-PCR	5'-GGTGGCATGATGCACTACAAG
AKIN10-F2	qRT-PCR, RT-PCR	5'-GCGCAGATGGTATGCTCAGT
AKIN10-R2	qRT-PCR, RT-PCR	5'-TTGACAACATTGGGCGACTT
AKIN10-F3	qRT-PCR, RT-PCR	5'-CGGGTACGAGAGATCCAGTAAT
AKIN11-F	qRT-PCR, RT-PCR	5′-AGCATGTTGTCACAGGGCAT
AKIN11-R	qRT-PCR, RT-PCR	5′-TGAGGATGCATAAACAACCGT
SUS4-F	qRT-PCR, RT-PCR	5′-ACCAAGACCTGGAGTTTGGG
SUS4-R	qRT-PCR, RT-PCR	5′-TCGACGAGCTCTTCCTTGAA
SUC2-F	qRT-PCR, RT-PCR	5′-ATCTGTGGGAGGTGGACCAT
SUC2-R	qRT-PCR, RT-PCR	5′-TAGCTTTGAAGGCAGGAGCA
SUC6-F	qRT-PCR, RT-PCR	5′-TAAGGCAGATTCCGACAACG
SUC6-R	qRT-PCR, RT-PCR	5′-AACCATGCGATCCAGTTCAA
SUC7-F	qRT-PCR, RT-PCR	5′-TAAGGCAGACTCCGACAACG
SUC7-R	qRT-PCR, RT-PCR	5′-AACCATGCGATCCAGTTCAA
SUC8-F	qRT-PCR, RT-PCR	5′-GCAGACTCGGACAACGAGAA
SUC8-R	qRT-PCR, RT-PCR	5′-AACCATGCGATCCAGTTCAA
AKIN10-BiFC-F	subcloning	5′-TAGAATTCCATGTTCAAACGAGTAGATGAGTTTA
AKIN10-BiFC-R	subcloning	5′-TCAGGATCCTGAGGACTCGGAGCTGAGCAAGAAA
AKIN11-BiFC-F	subcloning	5′-TTTGAATTCAATGGATCATTCATCAAATAGATTT
AKIN11-BiFC-R	subcloning	5′-TTGGATCCTGATCACACGAAGCTCTGTAAGAAAG
IDD8-BiFC-F	subcloning	5′-TTTGAATCCATGACAAGTGAAGTTCTTCAAACAA
IDD8-BiFC-R	subcloning	5′-TTAGGATCCTAATCCATCCATTGATAGACGATGG
AKIN10-F	subcloning	5′-AAAAAGCAGGCTTCATGTTCAAACGAGTAGATGA
AKIN10-R	subcloning	5′-AGAAAGCTGGGTTCAGAGGACTCGGAGCTGAGC
AKIN11-F	subcloning	5′-AAAAAGCAGGCTTAATGGATCATTCATCAAATAGATTTG
AKIN11-R	subcloning	5′-AGAAAGCTGGGTTCAGATCACACGAAGCTCTGT
IDD8-T98A-F	subcloning	5′-GTTGAAGCAGAGGGCAAGCAAAG
IDD8-T98A-R	subcloning	5′-CTCACTTCTTTGCTTGCCCTCT
IDD8-S178A-F	subcloning	5′-GGCACCATTTTCGCAAGGCG
IDD8-S178A-R	subcloning	5′-GCTGTCTCGCCTTGCGAAAAT
IDD8-S182A-F	subcloning	5′-CTCAAGGCGAGACGCCTTTATC
IDD8-S182A-R	subcloning	5′-GCTCTATGAGTGATAAAGGCGTCT

Additional file 11. Primers used.
F, forward primer; R, reverse primer.
